# Electrophysiological correlates of object-repetition effects: sLORETA imaging with 64-channel EEG and individual MRI

**DOI:** 10.1186/1471-2202-13-124

**Published:** 2012-10-17

**Authors:** Myung-Sun Kim, Kyoung-Mi Jang, Huije Che, Do-Won Kim, Chang-Hwan Im

**Affiliations:** 1 Department of Psychology, Sungshin Women’s University, Seoul, South Korea; 2 Department of Biomedical Engineering, Hanyang University, 17 Haengdang-dong, Seongdong-gu, Seoul 133-791, South Korea; 3 Department of Biomedical Engineering, Yonsei University, Wonju, South Korea

**Keywords:** Left superior temporal gyrus, Object categorization task, Object-repetition priming, Old/new effect, sLORETA

## Abstract

**Background:**

We investigated the electrophysiological correlates of object-repetition effects using an object categorization task, standardized low-resolution electromagnetic tomography (sLORETA), and individual magnetic resonance imaging. Sixteen healthy adults participated, and a total of 396 line drawings of living and non-living objects were used as stimuli. Of these stimuli, 274 were presented only once, and 122 were repeated after one to five intervening pictures. Participants were asked to categorize the objects as living or non-living things by pressing one of two buttons.

**Results:**

The old/new effect (i.e., a faster response time and more positive potentials in response to repeated stimuli than to stimuli initially presented) was observed at 350-550 ms post-stimulus. The distributions of cortical sources for the old and new stimuli were very similar at 250-650 ms after stimulus-onset. Activation in the right middle occipital gyrus/cuneus, right fusiform gyrus, left superior temporal gyrus, and right inferior frontal gyrus was significantly reduced in response to old compared with new stimuli at 250-350, 350-450, 450-550, and 550-650 ms after stimulus-onset, respectively. Priming in response time was correlated with the electrophysiological priming at left parietal area and repetition suppression at left superior temporal gyrus in 450-550 ms.

**Conclusions:**

These results suggest processing of repeated objects is facilitated by sharpening perceptual representation and by efficient detection or attentional control of repeated objects.

## Background

Priming has received a great deal of interest because it is one of the most basic forms of memory, influencing the perception and interpretation of the world [1]. Priming is increasingly accepted as a useful method for studying brain plasticity and its relationship to implicit learning [2]. A priming effect has been shown to occur during indirect memory tasks such as the categorization task (living vs. non-living objects) and the lexical decision task (words vs. nonwords). In the priming task, subjects responded more rapidly and accurately to previously experienced stimuli (old) than to stimuli presented for the first time (new) [3]. These increases in speed and accuracy of responses to old stimuli in these indirect memory tasks have been referred to as the repetition priming or repetition effect.

Neuroimaging studies have investigated the neurophysiological index of repetition priming using functional magnetic resonance imaging (fMRI) or positron emission tomography (PET). These studies found reduced activation in response to old stimuli relative to new ones, a phenomenon termed “repetition suppression” [1,4]. With respect to object-repetition priming derived from tasks such as the object categorization task, neuroimaging studies have reported that several brain areas, including the temporal/occipital areas and the inferior frontal regions, respond less strongly to old pictures of objects than to new ones [5-8]. Furthermore, recent studies attempting to elucidate whether repetition suppression forms the neural basis of behavioral priming by investigating the relationship between repetition suppression and behavioral priming have reported significant correlations. For example, Soldan et al. [8] observed correlations between repetition suppression in the bilateral fusiform gyrus and behavioral priming of familiar objects. Wig et al. [7] also investigated the relationship between repetition suppression and behavioral priming by using transcranial magnetic stimulation (TMS) to disrupt activity in the left frontal cortex during an object categorization task. Left-frontal TMS disrupted behavioral priming and repetition suppression in the left inferior frontal gyrus and lateral temporal cortex.

Results of neuroimaging studies regarding repetition suppression and its relationship to behavioral priming indicate that several, albeit not all, stages in the processing pathway between stimulus and response can be facilitated by repetition, and that not all brain regions showing repetition suppression contribute to behavioral priming [1]. Furthermore, these results indicate that in order to understand the neural substrates underlying repetition priming, it is necessary to investigate the temporal changes of cortical activations, which occur as the result of repetition. In other words, these results indicate the importance of investigating the spatiotemporal dynamics underlying repetition priming [9].

Event-related potentials (ERPs), the electrical activity time locked to external events, have been widely used to investigate repetition effects due to the high temporal resolution associated with this technique. The ERPs elicited by repeated stimuli show more positive potentials than those elicited by stimuli presented for the first time; this has been referred to as the old/new effect [10,11]. The old/new effect usually occurs between 300 and 600 ms after stimulus onset, elicited by common objects or faces [12,13] as well as by words [14-16]. Two components of ERPs are known to be sensitive to stimulus repetition: N400, which has been linked to semantic processing [17,18], and a late positive component (LPC: sometimes called P600) that has been linked to incidental recollection of previously studied items [19,20]. It has been suggested that the old/new effect results from attenuation of N400 and enhancement of the late positive component [21,22].

Although ERPs offer the temporal course of electrophysiological activities involved in repetition priming, traditional ERP analyses do not provide information about underlying sources of these electrophysiological activities due to the limited spatial resolution. The spatial resolution can be improved by the use of high-density electrode arrays, and a number of current-density estimation techniques have been developed to determine electrophysiological source locations. Low-resolution electromagnetic tomography (LORETA) [23] is one of the methods widely used for solving the inverse problem, because it does not require the assumption of a specific number of sources [24]. The assumption made in LORETA is that the inverse problem can be solved by determining the spatially smoothest current distribution, which is achieved by applying a Laplacian operator to the current density. By making this assumption, a particular current-density distribution can be obtained among the infinite number of solutions to the inverse problem.

Very few studies have investigated the sources of object-repetition priming using LORETA. For example, Guo et al. [12] investigated the sources of object-repetition priming by applying the LORETA method at the largest mean global field power (MGFP) of the difference in EEG waveforms between the first and the second presentations and observed repetition enhancement at the frontal region. However, the authors employed a delayed match-to-sample task rather than an implicit task. Additionally, they used a spherical head model, which can produce large localization errors due to the poor fit of sphere to the actual shape of a head.

The purpose of this study was to localize the generators of the object-repetition effects obtained in the implicit memory task. Particularly, we were interested in the spatiotemporal stages underlying visual object-repetition priming, i.e., when and where the repetition suppression occurs. In addition, we investigated whether repetition suppression is related to behavioral priming. For these purposes, standardized low-resolution electromagnetic tomography (sLORETA) incorporated in CURRY v.6.0 (Compumedics Ltd., Australia) was utilized, and each subject’s own MRI was used as a realistic head model of the boundary element method (BEM). Accurate electrode locations and differences in shape and size of individual brains could then be taken into account by utilizing the individual anatomical information, in order to enhance the reliability of source imaging results. To our knowledge, with the exception of a previous study performed by our lab that located the sources of the word-repetition effect [15], this is the first study to determine the cortical sources of the object-repetition effect using individual MRI and high-density EEG within the general framework of voxel-based statistical parametric mapping. To evaluate object-repetition effects, we recorded the ERPs resulting from an object categorization task in which line-drawings of objects were presented visually once or twice and participants were asked to determine whether each stimulus was living or non-living. The ERPs elicited by the old objects were compared with those elicited by the new objects, and the generators of object-repetition effects were examined by conducting a sLORETA analysis on the ERPs under each condition.

## Results

### Behavioral results

The statistical analysis of response times showed a main effect of old-new condition (*F*(1,15) = 29.2*, P* < .001). The old objects elicited significantly faster responses than did the new objects: mean RTs for old and new objects were 426 ms (SD = 14) and 453 ms (SD = 16), respectively. We found no significant differences between old and new objects in terms of error rates (*F*(1,15) = 1.9, *ns)*. The mean error rates for old and new objects were 4.1% (SD = 1.1) and 5.4% (SD = 1.7), respectively.

### Differences in mean amplitudes of old and new objects

**Figure 1:**
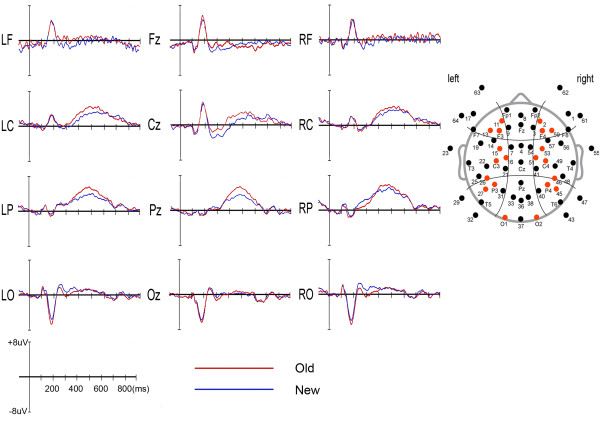
**Grand-average event-related potentials elicited by old and new objects at 4 midline sites and 4 regions of interest (ROIs) in left and right hemispheres, and the electrode sites included ROIs are designated by red dots (right panel).** LF: left frontal, RF: right frontal, LC: left central, RC: right central, LP: left parietal, RP: right parietal, LO: left occipital, RO: right occipital.

**Figure 2:**
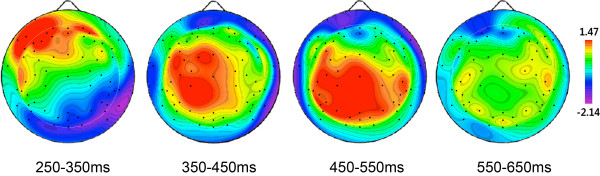
The topographies of the difference wave (ERPs elicited by old objects minus ERPs elicited by new objects) at 250-350, 350-450, 450-550, and 550-650 ms post-stimulus.

Figure 1 shows the grand-average ERP waveforms elicited by old and new objects at 4 midline sites and 4 regions of interest (ROIs) in left and right hemispheres. The old objects elicited more positivity than did the new objects particularly at the central and parietal recording sites. This object-repetition effect was observed at 350-550 ms post-stimulus. The topographical distributions of the difference between old and new objects at 4 time windows are presented in Figure 2.

Since the statistical analysis performed on 4 midline sites (Fz, Cz, Pz, and Oz) and 4 ROIs in left and right hemispheres yielded similar results for 250-650 ms intervals, we only report statistical results performed with 4 ROIs.

The statistical analysis for 250-350 ms bin showed no significant difference of old-new condition (*F*(1,15) = .4, *ns*). We observed a main effect of ROI (*F*(3,45) = 4.7, *P* < .05, ε = .56), with the largest and smallest amplitudes at the parietal and frontal sites, respectively. The mean amplitudes of left and right hemispheres did not differ (*F*(1,15) = .4, *ns*). In addition, an interaction effect of old-new condition and ROI was observed (*F*(3,45) = 6.1, *P* < .05, ε = .40). We performed separate ANOVAs for each condition, and we observed a main effect of ROI for new condition (*F*(3,45) = 11.2, *P* < .001, ε = .56), but not for old condition (*F*(3,45) = 1.9, *ns*). For new condition, amplitudes measured at the parietal site were significantly larger than frontal (*P* < .01), central (*P* < .01), and occipital (*P* < .05) sites. And the occipital site showed larger amplitudes than frontal (*P* < .05) and central (*P* < .05) sites. We further analyzed the interaction effect using paired t-tests for each ROI. We observed an old-new effect only at central site (*t*(15) = -2.1, *P* = .05). This result, along with grand average ERP waveform of Cz shown in Figure 1, seems to indicate the beginning of old-new effect in central site possibly due to reduced N400 amplitude to old stimuli compared to new stimuli. No interaction effects of old-new condition and lateralization (*F*(1,15) = 1.4, *ns*), ROI and lateralization (*F*(3,45) = .7, *ns*) and old-new condition, ROI and lateralization (*F*(3,45) = .6, *ns*) were observed.

We found main effects of old-new condition (*F*(1,15) = 8.4, *P* < .05, ε = 1.00) and ROI (*F*(3,45) = 17.7, *P* < .0001, ε = .64) for the 350-450 ms bin. The old stimuli elicited larger amplitudes than did the new stimuli. And the parietal site showed larger amplitudes than frontal (*P* < .0001), central (*P* < .0001) and occipital (*P* < .0001) sites, and the central site showed larger amplitudes than frontal site (*P* < .01). The main effects of old-new condition and ROI seem to reflect the beginning of LPC at this time interval, which could also be found in Figure 1. No lateralization effect was observed (*F*(1,15) = .1, *ns*). Interaction effects of old-new condition and ROI (*F*(3,45) = 2.0, *ns*), old-new condition and lateralization (*F*(1,15) = .2, *ns*), ROI and lateralization (*F*(3,45) = 1.0, *ns*), and old-new condition, ROI and lateralization (*F*(3,45) = .5, *ns*) were not statistically significant.

We observed significant differences in old-new condition (*F*(1,15) = 9.1, *P* < .01, ε = 1.00) and ROI (*F*(3,45) = 28.6, *P* < .0001, ε = .66) for the 450-550 ms bin. The old stimuli elicited larger amplitude than did the new stimuli. With regard to ROI, the parietal site showed larger amplitudes than frontal (*P* < .0001), central (*P* < .01) and occipital (*P* < .0001) sites, and the central site showed larger amplitudes than frontal (*P* < .0001) and occipital (*P* < .05) sites. The observed main effects of old-new condition and ROI seem to reflect the rise to peak of LPC at this time interval, which could also be found in Figure 1. No lateralization effect was observed (*F*(1,15) = .1, *ns*). Interactions of old-new condition and ROI (*F*(3,45) = 2.1, *ns*), old-new condition and lateralization (*F*(1,15) = .3, *ns*), ROI and lateralization (*F*(3,45) = .1, *ns*), and old-new condition, ROI and lateralization (*F*(3,45) = 1.4, *ns*) were not significant.

For the 550-650 ms bin, no significant difference of old-new condition (*F*(1,15) = .1, *ns*) was observed. However, we found a main effect of ROI (*F*(3,45) = 20.2, *P* < .0001, ε = .64). The central and parietal sites showed larger amplitudes than frontal (*P* < .0001) and occipital (*P* < .0001) sites, and the amplitudes between central and parietal sites were not significantly different (*P* = .82). As can be shown in Figure 1, these results, i.e., only ROI effect but no old-new effect, indicate the decrease and end of the late positive component at 550-650 ms bin. The mean amplitudes of left and right hemispheres did not differ (*F*(1,15) = .1, *ns*). No significant interaction effects of old-new condition and ROI (*F*(3,45) = .7, *ns*), old-new condition and lateralization (*F*(1,15) = .2, *ns*), ROI and lateralization (*F*(3,45) = .1, *ns*), and old-new condition, ROI and lateralization (*F*(3,45) = .6, *ns*) were observed.

### Source analysis

Based on the grand average difference mean global field power (old minus new) and visual inspection of individual participants’ mean global field power, maximum repetition priming at 250-350 ms (mean latency: 275 ms), 350-450 ms (mean latency: 392 ms), 450-550 ms (mean latency: 499 ms), and 550-650 ms (mean latency: 593 ms) were determined. sLORETA analysis was conducted individually at the time when the maximum repetition priming was observed, and individual participants’ peak time source images were included in one-sample and paired t-tests.

**Figure 3:**
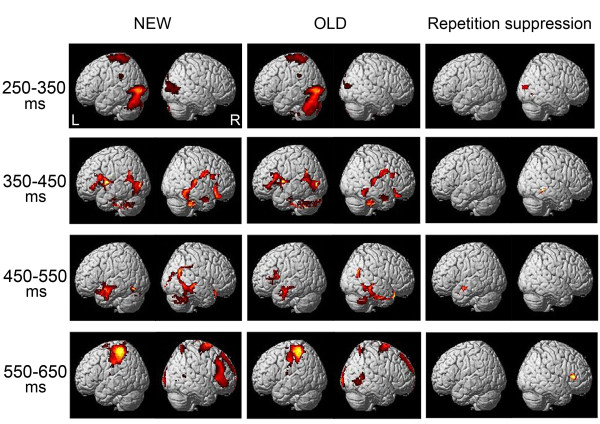
**ERP generation elicited by old and new objects at latency of 250-350, 350-450, 450-550, and 550-650 ms after stimulus onset.** Under the null hypothesis that there are no regional specific generators, the current density will be distributed around global mean by virtue of the global normalization. Under the alternate hypothesis of a regionally specific generator, the mean current density will be significantly different from global mean. These images were thresholded at t = 14.75 (*P* < 0.05 family wise error, extent *k* = 50). Right panel demonstrates reductions in cortical sources by old objects in the right middle occipital gyrus (BA 18, 19)/cuneus (BA 17), right fusiform gyrus (BA 37), left superior temporal gyrus (BA 22) and right inferior frontal gyrus (BA 45) at250-350, 350-450, 450-550, and 550-650 ms post-stimulus, respectively (t = 3.36, *P* < 0.005 uncorrected, extent *k* = 50). L/A: left anterior, R/P: right posterior.

Figure 3 (left and middle panel) shows the ERP generations elicited by new and old objects at 250-350, 350-450, 450-550 and 550-650 ms post-stimulus. This figure registers the statistic map threshold at t = 14.75 (*P <* .05 family wise error (FEW) corrected) with a contiguous 50-voxel extent (voxel size: 2.0 mm × 2.0 mm × 2.0 mm).

During 250-350 ms sources elicited by new objects were found at inferior occipital gyrus (BA 18, 19) and superior parietal lobule (BA 7) in the left hemisphere and at middle occipital gyrus (BA 18), and cuneus (BA 17) in the right hemisphere. Sourced elicited by old objects were found in the inferior occipital gyrus (BA 18, 19) and superior parietal lobule (BA 7) in the left hemisphere and at middle occipital gyrus (BA 18) and cuneus (BA 17) in the right hemisphere.

During 350-450 ms sources elicited by new objects were found at inferior occipital gyrus (BA 19), fusiform gyrus (BA 37), superior temporal gyrus (BA 39) and inferior frontal gyrus (BA 45) in the left hemisphere and at inferior occipital gyrus (BA 19), fusiform gyrus (BA 37) and inferior frontal gyrus (BA 44) in the right hemisphere. Sourced elicited by old objects were found in the inferior occipital gyrus (BA 19) and superior temporal gyrus (BA 39) and inferior frontal gyrus (BA 45) in the left hemisphere and at inferior occipital gyrus (BA 19), fusiform gyrus (BA 37) and inferior/middle frontal gyrus (BA 44/9) in the right hemisphere.

For 450-550 ms sources elicited by new objects were found at the fusiform gyrus (BA 37), middle/superior temporal gyrus (BA 21/22, 38) and inferior frontal gyrus (BA 44) in the left hemisphere and at the occipital lingual gyrus (BA 18), fusiform gyrus (BA 37), middle temporal gyrus (BA 21/39) and inferior frontal gyrus (BA 44) in the right hemisphere. Sources elicited by old objects were found at the middle/inferior frontal gyrus (BA 9/44) and the middle/superior temporal gyrus (BA 21/22) in the left hemisphere and at the fusiform gyrus (BA 37), middle/superior temporal gyrus (BA 21/22) and inferior frontal gyrus (BA 44) in the right hemisphere.

At 550-650 ms bin sourced elicited by new stimuli were observed at inferior/superior parietal lobule (BA 40/7), postcentral gyrus (BA 2, 3, 5) and middle frontal gyrus (BA 6) in the left hemisphere and at inferior temporal gyrus (BA 37), inferior/superior frontal gyrus (BA 45/6, 10) in the right hemisphere. Sources elicited by old objects were found at inferior/superior parietal lobule (BA 40/7), postcentral gyrus (BA 2) and middle frontal gyrus (BA 6) in the left hemisphere and at occipital lingual gyrus (BA 18), inferior temporal gyrus (BA 37), medial frontal gyrus (BA 9) and superior frontal gyrus (BA 6, 10) in the right hemisphere.

Figure 3 (right panel) demonstrates the statistically significant reduction in the current-density areas observed under the old condition compared with those observed under the new condition at 250-350, 350-450, 450-550 and 550-650 ms after stimulus-onset. The threshold of significance for the clusters was defined as containing at least 50 contiguous voxels exceeding a t value of 3.36, which corresponds to an uncorrected significance level of 0.005. The current density in the right middle occipital gyrus (BA 18, 19) and cuneus (BA 17) at 250-350 ms, in the right fusiform gyrus (BA 37) at 350-450 ms, in left superior temporal gyrus (BA 22) at 450-550 ms, and in the right inferior frontal gyrus (BA 45) at 550-650 ms under the old condition showed a statistically significant reduction compared with that under the new condition.

### Correlations between repetition suppression and behavioural priming

Behavioral priming was defined as the differences in mean response time and error rate between new and old stimuli, and electrophysiological priming and repetition suppression were calculated as the differences in mean amplitudes and current density between new and old stimuli. Priming in response time was significantly correlated with electrophysiological priming at left parietal site (*r* = .5, *P* < .05) and repetition suppression at left superior temporal gyrus (*r* = .5, *P* < .05) in 450-550 ms bin.

## Discussion

The brain activity underlying object-repetition priming using a categorization task was investigated in this study. In terms of behavioral data, old stimuli elicited more rapid responses than new stimuli in all participants. Additionally, old stimuli elicited more positive potentials than new stimuli at 350-550 ms after stimulus-onset. Taken together, these results indicate that repetition priming was observed both behaviorally and electrophysiologically.

According to the current results (Figure 3), a common network of brain regions was activated by both old and new stimuli during the categorization task. The sources elicited by old stimuli were very similar to those elicited by new stimuli at 250-650 ms after stimulus-onset, suggesting that the occipito-temporal [25,26] and prefrontal areas [27] may act as neural pathways underlying implicit object identification. Present result is consistent with previous findings that activation elicited by new and old stimuli is similar and repetition effect occurs only after the initial activation of the network [28].

The right middle occipital gyrus/cuneus and right fusiform gyrus were found to be significantly less active in response to old objects than to new objects at 250-350 and 350-450 ms after stimulus, respectively. This is consistent with results of previous EEG [29,30] and neuroimaging studies [5,8] that observed less activation in occipitotemporal areas in response to old stimuli than to new ones. For example, several EEG studies have reported an attenuation of induced *gamma*-band responses (iGBR), regarded as a signature of the memory trace of a stimulus, following repeatedly presented pictures of objects in posterior sites at 250-440 ms post-stimulus [29] or in the parieto-occipital sites at about 300-400 ms after stimulus onset [30]. In addition, Vuilleumier et al. [5] observed reduced activation in right occipitotemporal areas in response to repeated visual stimuli than to those presented for the first time, and Soldan et al. [8] found a significant correlation in repetition suppression in ventral visual areas including fusiform gyrus and behavioral priming. Since ventral visual system is involved in identification and discrimination of categorically related objects [31], repetition suppression in the middle occipital gyrus and fusiform gyrus in response to old stimuli than to new ones reflects facilitated perceptual processing of repeated stimuli.

The left superior temporal gyrus (STG) was also found to be significantly less active in response to old objects than to new objects at 450-550 ms after stimulus-onset, and repetition suppression in the left STG was significantly associated with priming in response time. Although the STG is known to be involved in several functions, including information integration [32] and processing of complex configurations [33], the STG seems to be involved in semantic processing [34] since significant repetition suppression in STG has been observed during a semantic priming task [35]. Furthermore, the STG has been reported as the source of the N400, which is one of ERP components contributing to the generation of repetition priming [36].

The functional significance of N400 is ambiguous [37], but the N400 is known to be involved in semantic processing, since N400 is sensitive to semantic deviation [38-41], and N400 amplitude is reduced when target stimulus is preceded by semantically related primes or by the same stimulus. For example, Race et al. [9] investigated whether different forms of learning are associated with repetition suppression in different cortical regions by manipulating repetition at three levels: stimulus, stimulus-decision and stimulus–response repetitions. They observed that the N400 amplitude decreased when stimuli were repeated, independent of stimulus-decision or stimulus–response repetition, compared with novel stimuli. Based on these results, they suggested that N400 priming effect reflects the strengthening of semantic representations that facilitate the “bottom-up” retrieval of relevant information when a previously processed stimulus is re-encountered.

These findings suggest that the STG is critical for sharpening the semantic representation of a stimulus. Thus, compared with a stimulus presented for the first time, a repeated stimulus would be processed more rapidly or efficiently, leading to a more rapid response to old stimulus and reduced activation of the STG in response to old stimulus.

We also found reduced activation in the right inferior frontal gyrus (RIFG) at 550-650 ms after stimulus-onset. The RIFG is known to be involved in several functions such as response inhibition, target detection or attentional control. For example, activation in RIFG is increased when a pre-potent response is required to be withheld [42], pre-learned target objects are detected [43], or attentional switching between tasks is required [44]. These findings suggest that reduced RIFG activation in response to old stimuli than to new stimuli reflects that re-exposure to stimuli could facilitate the detection and/or attentional control of these stimuli.

Several neuroimaging studies have reported that the left inferior frontal gyrus was less active in response to old objects than to new ones, and suppression in this area was correlated with behavioral priming [6,7]. Contrary to these results, repetition suppression in left prefrontal areas was not observed in the present study. These different results regarding cortical regions showing repetition suppression may be attributable to the different repetition intervals, number and type of stimulus repetition employed by the studies. The lags between initial and subsequent presentations of stimuli could have affected activations in response to the repeated stimuli [45]. Most fMRI studies that observed repetition suppression in the left inferior prefrontal cortex employed a paradigm that included a pre-scan study phase and a scan test phase in which the repetition of stimuli usually occurred several minutes after the initial presentation [46]. In contrast, the present study used relatively short repetition intervals (2.5-12.5 s). Schacter et al. [4] proposed that at least two distinct effects are involved in repetition priming: the “sharpening” or “tuning” effects, mediated predominantly by posterior areas such as the temporo-occipital areas that code for the perceptual representations of items [47], and the “synchronizing effect,” mediated by the inferior prefrontal regions that enable efficient processing and tighter coupling between stimulus and decision. It has been suggested that the left inferior prefrontal cortex may act as an executive system that mediates on-line retrieval of the long-term conceptual knowledge necessary for the repetition priming task [6,48]. Thus, the present results indicate that the repeated presentation of a stimulus within short intervals does not require this controlled processing, i.e., the on-line retrieval of long-term semantic knowledge, but requires only sharpening processing.

The number of stimulus-repetition could also have affected activations in response to the repeated stimuli. For example, Soldan et al. [8] observed activation in different cortical areas depending on the frequency of stimulus repetition; single repetition was correlated with suppression in bilateral fusiform gyrus, while multiple repetition of stimulus (3 times) was correlated with suppression in prefrontal and parietal areas in the same task. These results suggest that single and multiple repetition of stimulus facilitates perceptual processing and retrieval of semantic information about objects, respectively.

In addition, the left inferior frontal cortex is known as the source of LPC, which is one of ERP components contributing to the generation of repetition priming [36,49]. In the present study, we observed the largest positive peak at left parietal recording site in 450-550 ms post-stimulus (Figure 1), and the difference amplitude between old and new stimuli obtained at left parietal site in 450-550 ms bin was significantly associated with priming in response time. These results indicate that the LPC contributes to the generation of old/new effect. The LPC repetition effect is known to index recollection or retrieval of prior episode [50], but Race et al. [9] suggested that LPC repetition effect is not driven by retrieval of stimulus or stimulus–response but by retrieval of stimulus-decision associations, which serves to reduce decision uncertainty and facilitate selection of a response. Since the left inferior prefrontal area is involved in association between stimulus and decision [47,51] but the retrieval of stimulus-decision was not required in our study, the repetition suppression in left inferior frontal area and an association between the repetition suppression in this area and behavioral priming were not observed in the present study.

Our study has some limitations that should be addressed in future studies. First, the small sample size may limit the generalizability of the results about the neural correlates of object-repetition priming. Second, because it has been reported that distinct neural systems subserve the immediate and delayed repetition effect in explicit memory task [52], future studies that use only long-lag repetition intervals should be conducted. Third, because N400 and LPC reflect different aspects of repetition priming, future studies should use experimental designs evaluating N400 and LPC separately.

## Conclusions

In conclusion, old objects elicited significantly faster responses than new objects. The old objects also elicited more positive potentials than the new objects at 350-550 ms after stimulus onset. The distribution of cortical sources for old objects was similar to that for new objects. A significant reduction in activities in the right middle occipital gyrus/cuneus, right fusiform gyrus, left STG and RIFG was observed for old objects compared with new objects at 250-350, 350-450, 450-550 and 550-650 ms after stimulus onset, respectively. In addition, priming in response time was significantly associated with electrophysiological priming at left parietal site and repetition suppression at left STG in 450-550 ms after stimulus-onset. These results suggest that processing of repeated objects is facilitated by sharpening perceptual representation and by efficient detection or attentional control of repeated objects. In addition, these results provide valuable information about the spatiotemporal stages underlying the object-repetition priming, particularly when and where repetition suppression occurs, and how repetition suppression is related to behavioral priming.

## Methods

### Participants

Sixteen healthy right-handed college students (eight males, eight females) with a mean age of 24 years (SD: 2.4, age range: 20-28) participated in the experiment. Mean IQ of the participants evaluated by the Korean version of the Wechsler Adult Intelligence Scale [53] was 117 (SD: 10, range: 102-136). The Structured Clinical Interview for DSM-IV Non-patient [54] was administered to ensure that none of the participants had histories of psychiatric, medical, or neurological disorders or of drug/alcohol abuse. All participants reported that they had normal corrected-vision and no vision-related problems. The present study was approved by the Sungshin Women’s University Institutional Bioethics Review Board, and written informed consent was obtained from all participants after they were given a complete description about the intended study and use of their MRI images for publication. The participants were paid for their participation in the study.

### Object categorization task

A categorization task was administered in order to measure object-repetition priming. A total of 396 line-drawings of living and non-living objects were selected from Snodgrass and Vanderwart’s volume [55] and standardized pictures published by the International Picture Naming Project to be used as stimuli. The stimuli were arranged in two blocks, and each block consisted of 137 non-repeated stimuli and 61 stimuli that were repeated after one to 5 intervening pictures. The lag of repetition ranged from one to 5 items in order to increase the magnitude of behavioral and electrophysiological repetition effects [6].

Of the repeated stimuli, 17 depicted living objects and 44 showed non-living objects; of the non-repeated stimuli, 39 depicted living and 98 showed non-living objects. Participants were asked to judge whether the presented item was living or non-living and to respond to living things by pressing one response button with right/left hand and to non-living things by pressing another response button with left/right hand. The buttons assigned for the two responses were counterbalanced across participants.

The stimuli were presented in foveal vision for 500 ms on a computer monitor using E-PRIME (Psychology Software Tools, Inc., Sharpsburg, PA, USA), and each subtended a vertical visual angle of 4.52° - 4.62° and a horizontal visual angle of 4.52° - 4.61°. The distance between participants and computer monitor was 80 cm. We ensured that the distance for all participants was the same by fixing the position of the chair so that everyone sat at the identical location. The inter-stimulus interval was 2.5 s, and a crosshair (+) was shown on the monitor for 500 ms as a fixation point prior to stimulus presentation. A practice block of trials was administered to ensure that participants understood the task prior to the experimental session.

### EEG recording procedures

Electroencephalographic (EEG) activity was recorded using a 64-channel Geodesic Sensor Net connected to a 64-channel, high-input impedance amplifier (Net Amp 300: Electrical Geodesics, Eugene, OR, USA) in an electrically shielded and soundproofed experimental room. Each electrode was referenced to the Cz site, and individual electrodes were adjusted until impedances were less than 50 kΩ [56]. Eye movements and blinks were monitored with electrodes placed near the outer canthus and beneath the left eye.

During the experiment, EEG activity was recorded continuously using a 0.1-100 Hz analogue bandpass and a sampling rate of 250 Hz. After data collection was completed, the EEG was segmented into 1000 ms epochs (including a 100 ms pre-stimulus baseline) with respect to the event markers. The epochs that were contaminated by artifacts such as eye blinks and eye movements were rejected before averaging (the threshold for artifact rejection was ±70 μV). The EEG epochs were then averaged for each participant and each old-new condition. An average-reference transformation was used to minimize the effects of reference-site activity [57]. ERPs were baseline-corrected with respect to the 100 ms pre-stimulus recording interval and were digitally low-pass filtered at 30 Hz. Only epochs with correct responses were included in the statistical analysis. The means of the trials of new and old stimuli included in the final statistical analysis were 94 (SD: 7) and 92 (SD: 7), respectively, t(15) = 1.59, ns.

### Estimations of current densities using sLORETA

High-resolution T1-weighted MRIs were obtained from all participants using a Philips 3 T scanner (Philips Intera, Philips Medical System, Best, The Netherlands) with a SENSE head coil using a 3D T1-TFE sequence configured with the following acquisition parameters: axial acquisition with a 224 × 256 matrix; 220 mm field view; 0.98 × 0.98 × 1.2 mm^3^ voxels; TE 4.6 ms; TR 9.6 ms; flip angle 8° slice gap 0 mm; and 1 average per slice. The scalp location of each electrode was determined with a FASTRAK 3D-digitizer (Polhemus Inc., VT, USA). The electrode locations were imported into the Curry v. 6.0 software, where the MRIs and electrode locations of the participants were spatially co-registered for source localization. Three points, nasion and the left and right preauricular points, were used to match each sensor location with individual MRIs.

We used a three-compartment boundary element model (BEM). The resolutions of the meshes were set to 9, 8, and 6 mm for the skin, skull, and brain, respectively. Standard conductivity was used (0.33, 0.0042, and 0.33 for the brain fluid, skull, and skin, respectively). After segmenting the gray matter of the brain, a representation of the cortex excluding the brainstem and cerebellum was computed to limit the source space for the inverse solution. The number of source points used for the inverse solution averaged 4405 (SD = 399) and ranged from 3815 to 5112. We reconstructed the current-density distribution on each individual’s cortex using the sLORETA algorithm [58] implemented in CURRY v. 6.0, which evaluates statistical source images by performing an inverse weighting of the LORETA imaging results and their estimated variances. The regularization parameter was automatically determined by the *χ*^2^ criterion method implemented in CURRY. We conducted the sLORETA analysis with the ERP data obtained at the time points at which mean global field power (an average wave of the common average re-referenced data) of object- repetition effects peaked, i.e., 250-350, 350-450, 450-550 and 550-650 ms post-stimulus.

For the group analysis of individual current-density images, we used the statistical parametric mapping toolbox (SPM8) ( http://www.fil.ion.ucl.ac.uk/spm/) implemented in Matlab version 7.1 (Mathworks, USA). We applied realigning, co-registering, normalizing, and smoothing for the spatial preprocessing of the current-density image [59]. Finally, statistical parametric mapping with a paired *t-*tests was applied to the normalized current-density images using SPM8 to statistically compare the current density elicited by the old and new objects. For reference, ERP generators elicited by the old and new stimuli were investigated separately using a one-sample *t-*tests.

### Statistical analysis for the evaluation of the object-repetition effect

Based on visual inspection of grand-average and individual ERP waveforms, four 100 ms bins were selected covering the period from 250 to 650 ms after stimulus-onset (250-350, 350-450, 450-550, and 550-650 ms). The mean amplitude of each bin was calculated and analyzed by repeated measures analysis of variance (ANOVA). The initial statistical analysis was performed on 4 midline sites (Fz, Cz, Pz, Oz), and then on 4 regions of interest (ROI; frontal, central, parietal and occipital) in the left and right hemispheres. The old-new condition, ROI (frontal, central, parietal and occipital sites) and hemispheres (left and right hemispheres) were within-subjects factors. The Greenhouse-Geisser corrections for sphericity violations were employed when appropriate, and the corrected *p* value is reported [60]. The relationship between behavioral priming and repetition suppression was evaluated using Pearson’s correlation coefficient.

The behavioral data (i.e., response time and error rates) were also analyzed using repeated measures ANOVA with old-new condition as a within-subject factor.

## Competing interests

The authors declare that they have no competing interests.

## Authors’ contributions

M-SK developed the experimental task. K-MJ conducted the ERP experiment. HC and D-WK conducted the source analysis. C-HI designed the experiment. All authors read and approved the final manuscript.
